# Stabilized Conversion Efficiency and Dye-Sensitized Solar Cells from *Beta vulgaris* Pigment

**DOI:** 10.3390/ijms14024081

**Published:** 2013-02-18

**Authors:** Angel Ramon Hernández-Martínez, Miriam Estévez, Susana Vargas, Rogelio Rodríguez

**Affiliations:** 1 Centro de Física Aplicada y Tecnología Avanzada, Universidad Nacional Autónoma de México (National Autonomous University of Mexico), Campus Juriquilla, Boulevard Juriquilla No. 3001, Juriquilla, Qro. C.P. 76230, Mexico; E-Mails: miries@fata.unam.mx (M.E.); vmsu@unam.mx (S.V.); 2 Centro de Investigación y de Estudios Avanzados del IPN, Unidad Querétaro, Apartado Postal 1-798, Querétaro, Querétaro C.P. 76001, Mexico

**Keywords:** dye-sensitized solar cell, betalaine, Tetraethylorthosilicate, solar energy, dip coating

## Abstract

Dye-Sensitized Solar Cells (DSSCs), based on TiO_2_ and assembled using a dye from *Beta vulgaris* extract (BVE) with Tetraethylorthosilicate (TEOS), are reported. The dye BVE/TEOS increased its UV resistance, rendering an increase in the cell lifetime; the performance of these solar cells was compared to those prepared with BVE without TEOS. The efficiency η for the solar energy conversion was, for BVE and BVE/TEOS, of 0.89% ± 0.006% and 0.68% ± 0.006% with a current density Jsc of 2.71 ± 0.003 mA/cm^2^ and 2.08 ± 0.003 mA/cm^2^, respectively, using in both cases an irradiation of 100 mW/cm^2^ at 25 °C. The efficiency of the BVE solar cell dropped from 0.9 ± 0.006 to 0.85 ± 0.006 after 72 h of operation, whereas for the BVE/TEOS, the efficiency remained practically constant in the same period of time.

## 1. Introduction

The use of natural pigments as sensitizing dyes for the conversion of solar energy in electricity represents a very attractive alternative, due to significant benefits such as low cost, easy processing, environmental friendliness, low human toxicity [1,2], *etc*. Natural pigments extracted from fruits and vegetables [3–6], such as chlorophyll and anthocyanins, have been extensively investigated as Dye-Sensitized Solar Cells (DSSCs) sensitizers.

However, chlorophyll, with two maxima absorbance close to 420 nm and 660 nm [7], has the inherent limitations of weak absorption of blue wavelengths and low efficiencies (η) for DSSCs, for example 0.27% from China loropetal [8]. On the other hand, the absorption spectra of the anthocyanins, derived from cyanin in blackberries, which are responsible for the red and blue colors of many fruits and leaves, presents a more favorable overlap with the solar spectrum, thus producing efficiencies (η) of solar energy conversion close to 0.56% for DSSCs [9]. Recently, several studies have turned to using the flowering plants of the order Caryophyllales, which derive their colors from nitrogen-containing betalain pigments. Betalains have favorable light absorbing and antioxidant properties, and exist in nature, associated with various co-pigments that modify their light absorption properties, its functional groups (-COOH) bind with ease to the surface of TiO_2_ [10–15]. The efficiencies of solar energy conversion by betalains are 0.67% from beetroot (*Beta vulgaris*), compared to anthocyanins as dye sensitizers, slightly higher efficiencies (η) [6]. Nevertheless, this is rather low compared with efficiencies of 10 to 11% for a dye-sensitized based on ruthenium [2,16–20], one of the key of this disadvantage is the ratio of the rates of injection and recombination electron transfer [9,16,21], other parameter key is the rate at which the oxidized dye reacts with the reduced form of the redox mediator, which is typically I^−^/I_3_^−^ [6]. In general the lifetime for DSSCs is shorter than that of silicon cells, but synthetic dyes based on ruthenium compounds have a relatively long lifetime, corresponding with the natural dyes, which is an additional disadvantage. DSSCs’ lifetime depends mostly on solvent evaporation and high dye photo-reactivity resulting in a fast degradation of dye. To overcome the solvent evaporation problem, some authors have been working to improve the electrolyte and cell encapsulation [1,2]; for the second problem and specifically by betalains, this present work proposed the use of Tetraethylorthosilicate as photodegradation inhibiting agent to decrease the UV degradation with a concomitant increment in the lifetime of the cell.

## 2. Results and Discussion

### 2.1. Absorption Spectra

**Figure 1:**
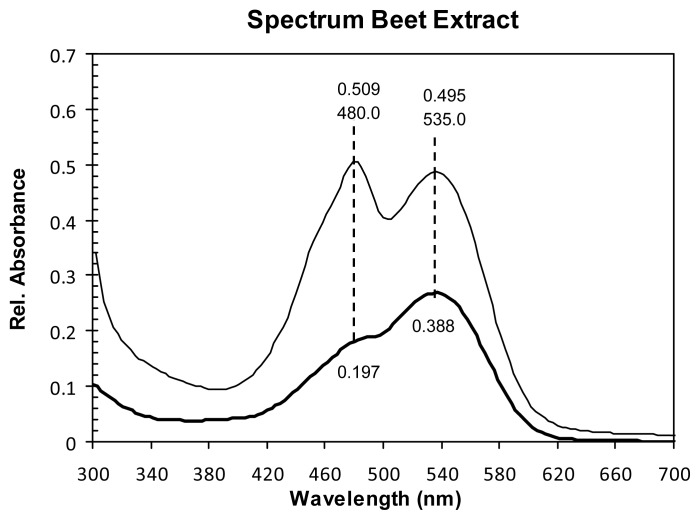
Absorption spectra of *Beta vulgaris* (BVE)/Tetraethylorthosilicate (TEOS) (thick line) and BVE (thin line), both diluted in water at pH = 3.5.

Absorption spectra of the BVE and BVE/TEOS were analyzed, both diluted in water at pH = 3.5, with the spectrophotometer at wavelengths between 260 and 800 nm, but only showing the area of interest, between 300 and 700 nm. In both cases, two peaks were found: one around 480 nm associated with the presence of betaxanthins, while the second one at 535 nm is attributed to the betanin. Absorbance peaks at 300 nm and 535 nm are characteristic for red violet betalain group, betacyanin [22], while for betaxanthins the characteristic peaks are at 280 nm and 480 nm [23]. As can be seen in Figure 1, the TEOS addition in the dye absorbance decreases 22% and 61% for betanin and betaxanthins respectively; in both cases, the solutions were prepared with 79.1 mg of powder dye in 50 mL of water, as explained in Section 3.2.

**Figure 2:**
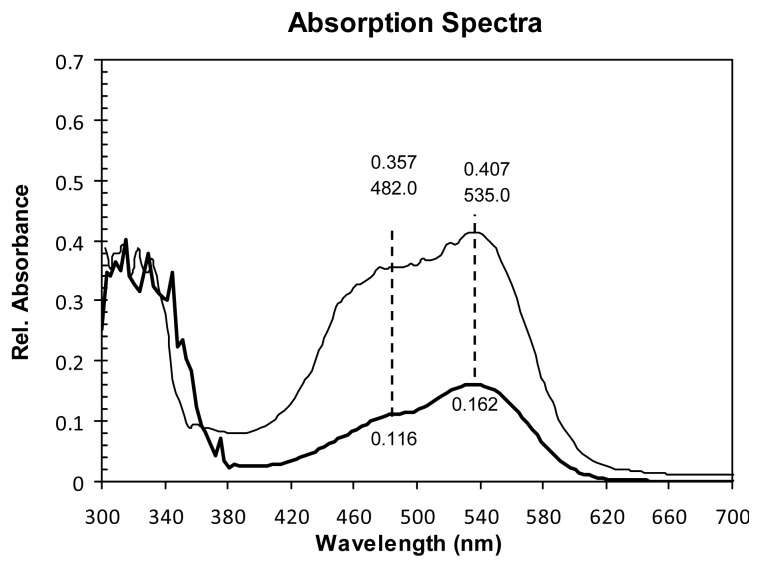
Absorption spectra of the photoanode: BVE/TEOS (thick line) and BVE (thin line), both dipped in a sol-gel solution during 48 h at room temperature.

Some electrodes were left immersed for 48 h in BVE, while others were immersed during the same period of time in the corresponding BVE/TEOS. It was found that there was not a significant increase in the absorption of the dye after 48 h. UV spectra of the electrodes after 48 h immersed in the dyes are shown in Figure 2, where it can be seen that the modified extract is less adsorbed than the natural extract of beetroot. This can be explained, as the number of hydroxyl groups of the carboxylic group are increased by the reaction with the silicon alkoxide; the other reason is that the absorption of the UV radiation produced by the silica itself.

We have not elucidated completely the reaction mechanism between the BVE and the TEOS in an aqueous solution; however, we believe that there is a chemical change in the BVE molecule produced by the reaction with TEOS: Based on the chemical reactivities of the functional groups and the organic chemistry theory, it is possible to explain the possible modification of the BVE: TEOS and BVE are together in an acidic medium at pH 5.5; thus, at least two simultaneous reactions take place: the hydrolysis reaction of TEOS and acid catalyzed esterification of the carboxyl groups of BVE.

**Scheme 1:**
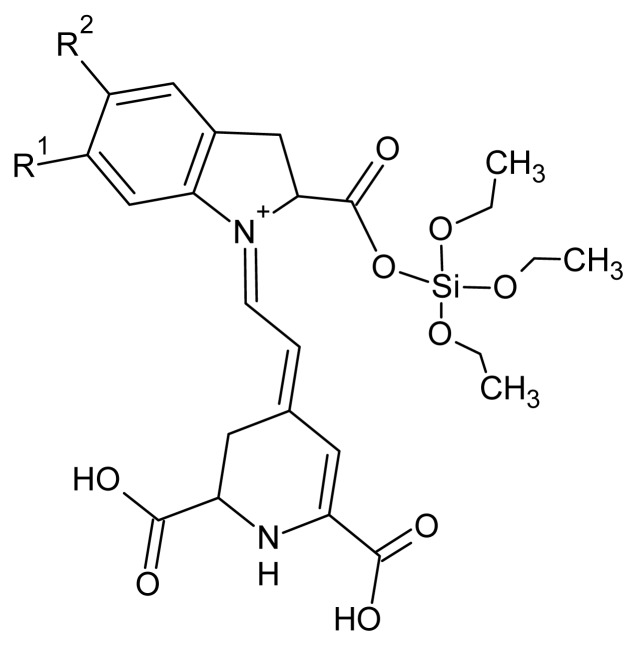
Graphic representation of the BVE/TEOS dye.

For the esterification, the first step corresponds to the protonation of the carboxyl groups present in the BVE molecule, with the consequent formation of water molecules, which are good leaving groups. In the second step, it is possible that the nucleophile, Si-(OCH_2_CH_3_)_4_, attacks the carbonyl cation, forming new bonds carbonyl-oxygen-silicon, which is schematically shown in Scheme 1.

### 2.2. Structure and Surface Characterization

**Figure 3:**
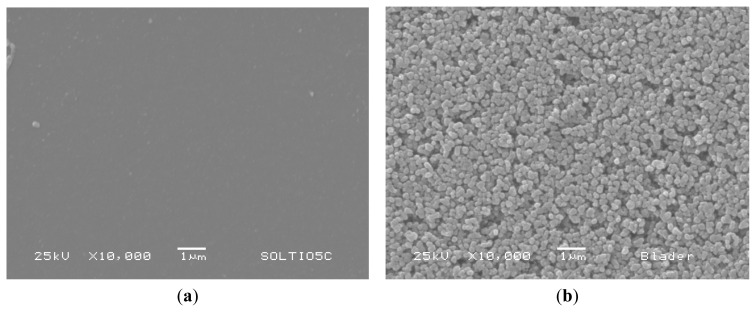
SEM images of (**a**) TiO_2_ compact layer overlying conductive glass plates and (**b**) the second nanoporous TiO_2_ layer produced by screen-printing technique.

The particle size distribution of the commercial TiO_2_ particles was determined resulting in 285 ± 15 nm. The TiO_2_ surface morphology is shown in Figure 3. Figure 3a shows a compact (pore-free) structure of the first thin layer of TiO_2_ obtained by immersing the ITO plates in a titanium alkoxide sol-gel precursor. This thin film acts as a blocking layer between the ITO layer and the dye-activated porous TiO_2_ layer. Calogero *et al.* [1] have proved the usefulness of the compact layer to achieve a higher efficiency in the performance of the DSSC. On the other side, Figure 3b shows the surface of the thick TiO_2_ coating obtained by the screen-printing technique. Here, it is possible to see a homogeneous mesoporous surface formed by the TiO_2_ nanoparticles. The nanoporous structure is useful because it has a high effective surface area to which the molecules of *Beta vulgaris* extracts can be linked.

### 2.3. Photoelectrochemistry

Both, BVE and BVE/TEOS, were used as dyes for solar cells. The short-circuit photocurrent density J_SC_, the current density at maximum power J_MP_, the open-circuit voltage V_OC_, the maximum power voltage V_MP_, the maximum power P_M_, the theoretical power P_T_, the fill factor FF, and the energy conversion efficiency η, were measured five times and are reported in Table 1.

**Figure 4:**
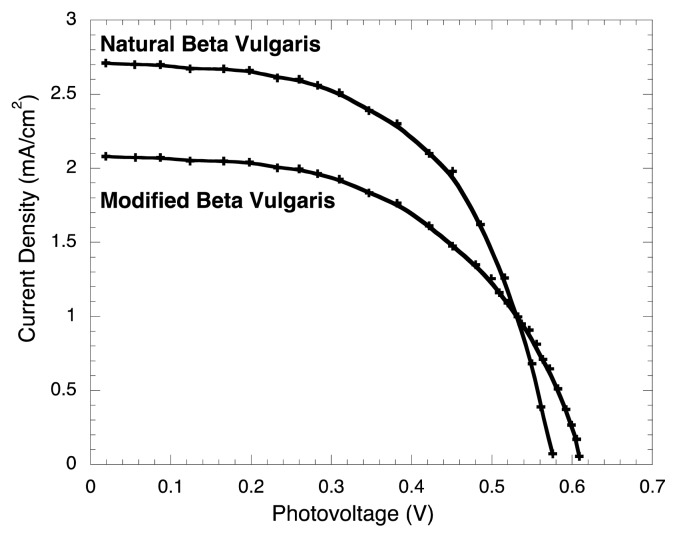
Photocurrent-photovoltage curves for BVE and BVE/TEOS solar cells.

**Figure 5:**
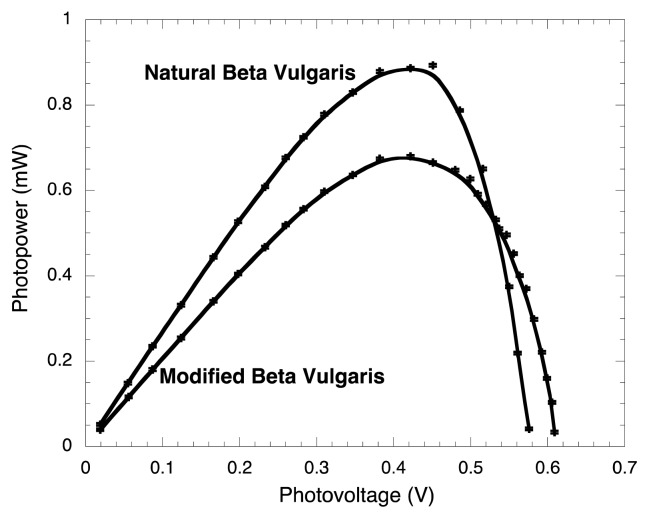
Power-photovoltage curves for BVE and BVE/TEOS solar cells.

It is important to conduct error analysis showing the errors in the experimental values and the propagated errors in calculated values based on experimental data. In this way, to validate the experimental data of current density and voltage, the statistical experimental errors and their propagation were determined for J_SC_, J_MP_, V_OC_, V_MP_, P_M_, FF, and the energy conversion efficiency η. Five different similarly prepared experiments were performed to determine the statistical error of voltage and current measurements. In Figures 4 and 5, every point is the average of five measurements, and the error bars are the standard deviation (δ) calculated for every point: 19 for un-modified *Beta vulgaris* and 27 for modified *Beta vulgaris* dyes; the error are also reported in the Table 1.

Error propagation (±Δ) was calculated according to the following general formula:

(1) $$\Delta f=\pm {\left|\sum_{i}^{n}\left|\frac{\partial f}{\partial X_{i}}\right|*\Delta X_{i}\right|}_{;}\;\;\;f=f(X_{1},X_{2},\ldots X_{i})$$

For the power:

(2) ΔP=±|J*ΔJ+V*ΔV|

For the efficiency:

(3) $$\Delta \eta =\pm \left|\Delta P_{m}\right|$$

For the filling factor:

(4) $$\Delta FF=\pm \left|\frac{P_{m}}{P_{T}^{2}}*\Delta P_{T}+\frac{\Delta P_{m}}{P_{T}}\right|$$

Consistent and comparable results were obtained for BVE compared to those obtained by Zhang *et al.* [6]. It was found that the J_SC_ using BVE/TEOS was 0.63 mA/cm^2^ lower than the corresponding value of BVE, as shown in Figure 4. This could be simply explained, as the photon-absorption of the un-modified dye is higher compared to the modified dye (see Figure 2). Figure 5 shows the power curves obtained as a function of voltage. It is possible to observe here that the P_M_ of BVE/TEOS solar cell was 0.21 mW lower than the corresponding value for BVE.

### 2.4. Stability Test

**Figure 6:**
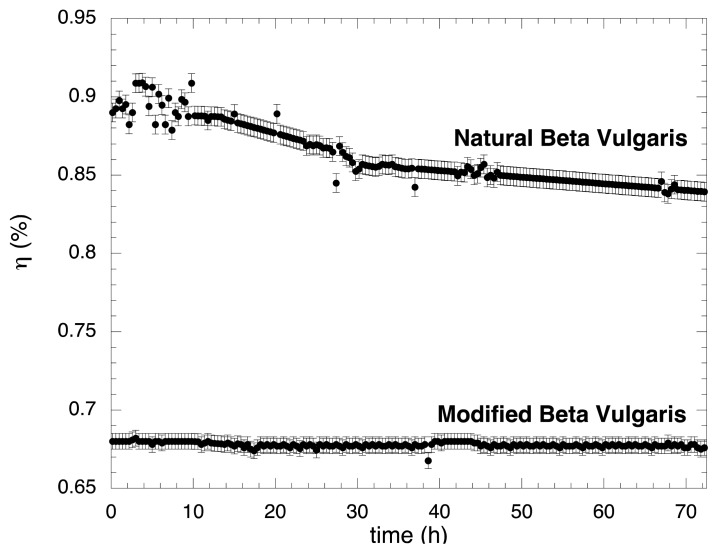
Time dependence of the efficiency for BVE and BVE/TEOS solar cells.

Tests on the stability of BVE and BVE/TEOS dyes were carried out by monitoring some indicative parameters, such as J_SC_ and η, under continuous sunlight (100 mW/cm^2^ and air mass 1.5) in a hermetically sealed solar cell with electrolyte solution at 25 °C during 72 h; the results are reported in Figure 6. Here it is possible to see that no significant changes were observed for BVE/TEOS during this period. However, for the BVE the efficiency was reduced as a function of time: a reduction of 6% was observed in this period. Assuming that the efficiency is reduced exponentially with time, solar cells fabricated with the BVE/TEOS have relaxation times over twenty times larger compared to the BVE ones. This means that the lifetime of the cells can be significantly increased when BVE/TEOS are used.

Photo-anodes with one porous layer of TiO_2_, manufactured by sol-gel, soaked for 12 h in HCl, immersed in the dye solutions for two nights at room temperature, washed with distilled water and ethanol and dried, were placed under the same conditions of illumination (100 mW/cm^2^) every four h; UV-Vis absorption spectrum was obtained after 120 h of irradiation.

**Figure 7:**
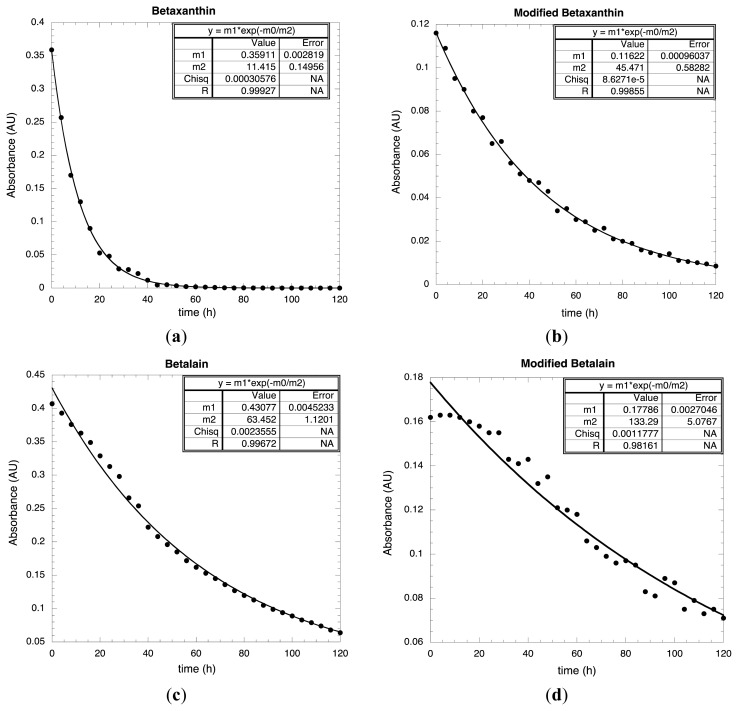
Absorbance *versus* Time for (**a**) BVE at 482 nm, (**b**) BVE/TEOS at 482 nm, (**c**) BVE at 535 nm and (**d**) BVE/TEOS at 535 nm.

From the UV-Vis spectra, it was possible to monitoring the stability of the following betaxanthin and betalain absorbance value at 482 nm and 535 nm respectively (see Figure 2). In Figure 7, it is possible to see the absorbance profile for unmodified betaxanthin. As shown, the behavior corresponds to a single exponential function where the relaxation time is 11.42 h (Figure 7a). As shown, for betaxanthin the relaxation time was increased from 11.42 to 45.47 h, *i.e.*, by a factor of 4, when the betaxanthin was chemically modified. It is possible to conclude that the chemical modification has an important stabilizing effect compared to the degradation suffered by the betaxanthin when was light irradiated.

In Figure 7c, the absorbance for the unmodified betalain is reported; the continuous line corresponds to a fitting using a single exponential function. For this sample, the relaxation time is 63.45 h. For this unmodified dye, it is possible to see the onset of two different regimes: from 0 to 30 h and from 40 to 120 h. However, the temporal behavior of these regimes is closely similar to each other. The chemical modification increases significantly the relaxation times for these regimes.

In Figure 7d, it is possible to observe the absorbance for the modified Betalain. All these data points were fitted using a single exponential function, producing a relaxation time of 133.3 h. However, it is possible to observe that there are two well defined regimes at the times 0–28 h and 32–120 h; these are characterized by a decay exponential behavior, but with different relaxation times each one.

**Figure 8:**
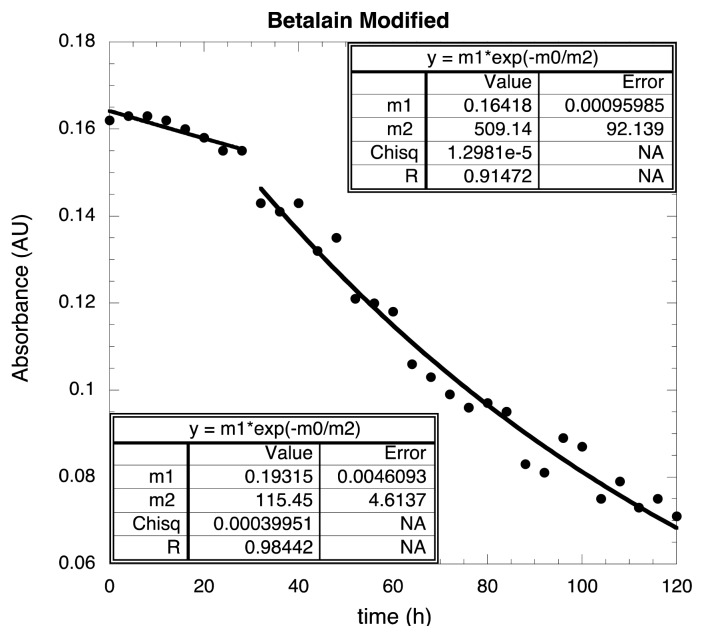
Detail of the Figure 7d for modified Betalain when the experimental data was fitted using a single exponential function for each of the two regimes: from 0 to 28 h and from 32 to 120 h.

In Figure 8, these two regimes were fitted separately using a single exponential for each one; the continuous lines correspond to the fitting. The relaxation times are 509.14 h and 115.45 h. This effect is obviously produced by the chemical modification. The modification stabilizes the dye, keeping the absorbance during the first 28 h practically constant; after this time, the absorbance is reduced at the same rate as the unmodified dye; this is the way the chemical modification stabilizes the dye. This is an important finding because, besides the low cost of the DSSCs, an increment in the lifetime positions of these solar cells in a more competitive scenario with respect to the inorganic-based solar cells was observed.

## 3. Experimental Section

### 3.1. Materials

The pigment was obtained using fresh beetroots (*Beta vulgaris* L. *Var Rubra*), Centrosperma species of the Chenopodiaceae family, collected from central Mexico. Tetraethylorthosilicate (TEOS), titanium isopropoxide, nitric acid, iodine, lithium iodide, 3-methoxypropionitrile, acetonitrile polyethylene glycol, titanium oxide (TiO_2_) nano-powder (mesh 320) and the solvents, and ITO-coated glass were supplied from Aldrich Chem. Co. (Toluca, Estado de México, México) and used as received. Ammonium acetate, ethanol, isopropanol, chloride acid, and ascorbic acid were supplied from J.T. Baker and used as received, as well. The ion exchange resin, Amberlite, was used to purify the pigment using a packed column. Silica nano-particles of 17 nm known as Aerosil R972 (Degussa, Germany), were suspended in water under strong agitation.

### 3.2. Preparation of BVE/TEOS

The beetroots were weighed without stems, washed, and placed in boiling water (98 °C) for two min to inactivate enzymes and increase the fixation in the beetroot. Later, they were cut and the juice was immediately extracted by mechanical action using an Omega juice extractor. The solids and large aggregates were removed from the juice, prior to the stabilization process, by filtering using a mesh sieve and by centrifugation at 13,000 rpm for 20 min.

The stabilization method consisted of two consecutive reactions: first, 100 mL of *BVE*, previously filtered and centrifuged, was placed into a ball flask. 19.6 mL of TEOS was poured to the juice with strong agitation for 1 h at room temperature. This mixture was introduced into the packed column containing the ionic resin (Amberlite) in order to remove all free sugar from the BVE. The flow rate through the column was 32 mL/min (20 BVE/h) and an aqueous solution 1 M of ammonium acetate at pH 5.5 was used as eluent; ascorbic acid was used to adjust the pH. 100 mL of solution collected from the column were added to a second ball flask and mixed with 19.6 mL of TEOS also under the same conditions. The product was filtered and lyophilized; an aqueous dispersion of 1 wt% of silica nano-particles with ascorbic acid at 0.1 wt%, was also used during the lyophilization process. Subsequently the BVE/TEOS was prepared from the lyophilized powder to be used in the solar cell: 79.1 mg of modified-dye sensitizer powder was dissolved in 25 mL of distilled water; the pH was adjusted to 3.5 by the addition of an aqueous solution 1N of HCl.

### 3.3. Electrodes Preparation

The conductive glass plates were ITO-coated glass slides (In_2_O_3_:SnO_2_) with nominal sheet resistance of 30–60 Ω/sq, 84% transmittance at 550 nm and dimensions (L,W,T) of 25 × 25 × 1.1 mm. The ITO substrates were ultrasonically cleaned in an ethanol-water mixture for 30 min and then heated at 450 °C during 30 min prior to put any layer. The photo-anodes were prepared by depositing two TiO_2_ films on the cleaned ITO glass: for the first one the ITO glass was immersed in a sol-gel solution containing a mixture of titanium isopropoxide, water and isopropanol at concentration 2:1:25 vol; the immersion was at constant speed (1.5 cm/min) [24]; the coated glass was heated at 450 °C during 30 min producing a densified TiO_2_ thin film with thickness of 90 nm; this film isolate the dye-activated porous TiO_2_ layer from the conductor glass. Subsequently, two edges of the ITO glass plate were covered with adhesive tape (Scotch 3 M) as spacers to control the thickness of the film; finally, a TiO_2_ paste was spread uniformly on the substrate by sliding a glass rod along the tape spacers. The TiO_2_ paste was prepared by mixing 3.0 g of TiO_2_ nano-powder, 10 mL of nitric acid 0.1 N and 4 mL of polyethylene glycol; this suspension was stirred in a closed glass container for 24 h to obtain a smooth paste with the appropriate viscosity. The film was heated at 500 °C for 60 min resulting in a mesoporous film with a thickness of around 8–10 μm and opaque. The TiO_2_ photo-anodes were first soaked for 12 h in HCl and then immersed in the dye solutions for two nights at room temperature, according to previously published procedures [6]. Later, the photo-anodes were rinsed with distilled water and ethanol and dried. Carbon coated counter electrodes were prepared according to a procedure reported elsewhere [25].

### 3.4. DSSC Assembling

The electrolyte solution was prepared as reported elsewhere [6]: 0.1 M of I_2_ was mixed with 0.05 M of LiI and 0.05 M of 3-methoxypropionitrile in 50 mL of acetonitrile (C_2_H_3_N) and stirring for 60 min. This electrolyte solution was poured in the mesoporous TiO_2_ film that was previously prepared using paraffin-film as framework to seal the cells to prevent liquids’ evaporation. The counter electrode was pressed against the impregnated anode and clamped firmly in a sandwich configuration. No leaks (solvent evaporation) were detected during the tests.

### 3.5. Characterization Techniques

The topography of the TiO_2_ films was obtained by using a scanning electron microscope (SEM) JEOL JSM-6060LV operated at 20 kV in secondary electron mode with different magnifications; the samples were previously coated with a gold film of around 50 nm. The particle size and the particle size distribution of the commercial TiO_2_ particles were determined using a light scattering apparatus, Brookhaven Instruments model BI200SM, equipped with a high speed digital correlator PCI-BI9000AT, a solid-state photon detector and a He-Ne laser of 35 mW Melles Griot 9167EB-1 as a light source. The crystalline structure of the TiO_2_ thin film was determined using a diffractometer Rigaku model Miniflex+ equipped with a radiation source of 1.54 Å and the angle 2θ was varied from 5° to 80° at a scan rate of 2°/min. Absorption spectra were obtained using a UV-Vis spectrometer Genesys 2PC. The DSSCs were illuminated using a 75 W halogen lamp with an incident power of about 100 mW/cm^2^ in an illumination area of 0.16 cm^2^; UV and IR filter glasses were used in front of the sample. Photocurrent and photovoltage were measured using a Keithley 2400 source meter; source voltage from 51 × 10^−6^ to 210 V, source current from 5 × 10^−11^ to 1.05 A, measure voltage from 1 × 10^−6^ to 211 V, and measure current from 1 × 10^−11^ to 1.055 A. The incident light power was determined using a Powermeter Thor Labs S130A (0–300 mw) and a Spectrometer Ocean Optics HR 400.

## 4. Conclusions

BVE and BVE/TEOS were studied as dyes for DSSCs. The results obtained for BVE dye are consistent with results previously reported. A significant high cell stability was found using BVE/TEOS dye, compared to the BVE dye; this provides solar cells with large lifetimes. This stability was demonstrated by measuring the efficiency during 72 h of continuous operation; the BVE/TEOS dye presented minor variations in efficiency (of the order of 0.08%), which is significantly smaller as compared with a variation of 6% for the BVE dye. This improvement in stability was achieved at the expense of a moderate reduction in J_SC_, FF, and η. Thus, the fabrication of solar cells using BVE/TEOS dye is a promising alternative for producing sensitized solar cells with improved lifetimes, making these more competitive compared to the inorganic-based solar cells.

## Figures and Tables

**Table 1:** Electrical characterization of all prepared solar cells.

	Jsc (mA/cm^2^)	J_MP_ (mA/cm^2^)	V_OC_ (V)	V_MP_ (V)	P_M_ (mW)	P_T_ (mW)	FF	η (%)
BVE	2.71 ± 0.003	1.98 ± 0.003	0.576 ± 0.0002	0.451 ± 0.0002	0.89 ± 0.006	1.561 ± 0.007	0.572 ± 0.003	0.89 ± 0.006
BVE/TEOS	2.08 ± 0.003	1.61 ± 0.003	0.609 ± 0.0002	0.422 ± 0.0002	0.68 ± 0.005	1.266 ± 0.006	0.537 ± 0.003	0.68 ± 0.005
